# Continuation of Pediatric Care after Transfer to Adult Care Among Autistic Youth Overlap of Pediatric and Adult Care

**DOI:** 10.1007/s10803-024-06314-5

**Published:** 2024-03-23

**Authors:** Joseph Sirrianni, Christopher Hanks, Steve Rust, Laura C. Hart

**Affiliations:** 1 Nationwide Children’s Hospital, 700 Children’s Drive, Columbus, OH 43205 USA; 2 The Ohio State University College of Medicine, Columbus, OH USA

**Keywords:** Autism, Transition to adult health care, Health transition, Adolescent, Young adult

## Abstract

The transition from pediatric to adult health care is a vulnerable time period for autistic adolescents and young adults (AYA) and for some autistic AYA may include a period of receiving care in both the pediatric and adult health systems. We sought to assess the proportion of autistic AYA who continued to use pediatric health services after their first adult primary care appointment and to identify factors associated with continued pediatric contact. We analyzed electronic medical record (EMR) data from a cohort of autistic AYA seen in a primary-care-based program for autistic people. Using logistic and linear regression, we assessed the relationship between eight patient characteristics and (1) the odds of a patient having ANY pediatric visits after their first adult appointment and (2) the number of pediatric visits among those with at least one pediatric visit. The cohort included 230 autistic AYA, who were mostly white (68%), mostly male (82%), with a mean age of 19.4 years at the time of their last pediatric visit before entering adult care. The majority (*n* = 149; 65%) had pediatric contact after the first adult visit. Younger age at the time of the first adult visit and more pediatric visits prior to the first adult visit were associated with continued pediatric contact. In this cohort of autistic AYA, most patients had contact with the pediatric system after their first adult primary care appointment.

## Introduction

The prevalence of autism is increasing (Maenner et al., 2020), with a growing population of autistic adolescents reaching adulthood (Van Naarden Braun et al., 2015). This demographic shift has sparked heightened interest in understanding the healthcare utilization patterns and experiences of autistic adolescents and young adults (AYA). Weiss et al. (2018) found that autistic youth ages 18 to 24 are more likely to visit their pediatrician compared to youth with other developmental disabilities and to youth without a disability. Ames et al. (2021) found that autistic AYA ages 14 to 25 had higher odds of receiving mental health care than AYA with attention-deficit hyperactivity disorder (ADHD) (OR 1.73, 95% CI 1.60–1.86) and AYA generally (OR 10.87, 95% CI 9.90–11.94). Zerbo et al. (2019) also found that autistic young adults ages 18 to 24 had higher odds of receiving mental health care (OR 1.9, 95% CI 1.7–2.2) and speech therapy (OR 7.5, 95% CI 2.4–23.8) than young adults ages 18 to 24 with ADHD. Autistic AYA have higher odds of presenting to the emergency department for psychiatric reasons compared to their non-autistic counterparts (Iannuzzi et al., 2022; Vohra et al., 2016).

However, not all service use was higher for autistic young adults. Zerbo et al. (2019) study found that autistic young adults were less likely to receive physical or occupational therapy than young adults with ADHD (OR 0.4, 95% CI 0.3–0.5) or young adults generally (OR 0.4, 95% CI 0.3–0.6). Furthermore, autistic women were found to be less likely to undergo cervical cancer screening than young adults with ADHD or young adults generally.

Studies also suggest that use of health care by autistic AYA varies by age. For example, adolescents ages 14 to 17 with ADHD were more likely to be prescribed psychotherapeutic medications than autistic adolescents ages 14 to 17, but autistic young adults ages 18 to 25 were more likely to be prescribed psychotherapeutic medications than young adults ages 18 to 25 with ADHD (Ames et al., 2021). In other work that looked at changes in health care utilization among autistic youth (ages 16 to 20), older autistic youth were more likely to see a neurologist and be admitted to the hospital for a psychiatric problem, but less likely to be admitted to the hospital overall when compared to younger autistic youth (Tunesi et al., 2019).

These variations in care use among age groups have implications for the transition from pediatric to adult health care for autistic AYA, defined as “the process of moving from a child to an adult model of health care with or without a transfer to a new clinician” (White & Cooley, 2018). In the United States, both expert opinion and current guidelines recommend a flexible approach to the transition process where the decision to transfer to a new provider is made when patients, families, and providers are ready, rather than being based strictly on age (Hardin & Hackell, 2017; Schor, 2015; White & Cooley, 2018).

Despite the recommendation for a flexible approach, there are several barriers and challenges that hinder a smooth transition for autistic AYA. Studies show that guidelines are not being consistently implemented, and that AYA are not getting appropriate transition support (Javalkar et al., 2022), and that autistic adolescents get less transition support than AYA without a developmental disability (Leeb et al., 2020). Surveys suggest that only one-third of autistic AYA had discussed transition with their pediatric medical providers (Cheak-Zamora et al., 2014). A separate study demonstrated that time limitations, lack of comfort or knowledge with the topic, or not knowing the patient or family well often led to not addressing transition-related topics during a visit or starting these conversations after the age of 18 (Harris et al., 2021). Parents report difficulty breaking bonds with pediatric medical providers (Cheak-Zamora & Teti, 2015; Cheak-Zamora et al., 2017). Additionally, adult medical providers often lack experience and comfort in caring for autistic adults (Cheak-Zamora & Teti, 2015; Cheak-Zamora et al., 2017; Mazurek et al., 2021; Nehring et al., 2015). All of these barriers, combined with the decreased availability of services as individuals age out of child and adolescent systems (Mauch et al., 2011), create many challenges that impact decisions about how and where to receive medical care during the transition period. With all these factors, it is not surprising that autistic adults report significant unmet healthcare needs (Nicolaidis et al., 2013), nor that their caregivers also feel that health care services for autistic adults are inadequate (Mazurek et al., 2021).

The recommendation for a flexible approach to the transition process creates a theoretical possibility that autistic AYA continue to access pediatric services for some care for a period of time after they start using adult services for other aspects of care. However, potential overlap that is theoretically possible has not been consistently accounted for in the approach to transition to adult health care. Some studies even define “successful transition” as the prevention of patients returning to pediatric care after the first adult appointment (known as a “bounce back”) (Gray et al., 2019; Sadun et al., 2022; Sharma & Sharma, 2022; Zupanc, 2020). To begin to resolve this discrepancy between the recommendations from guidelines and the current state of clinical care, it is crucial to have a thorough understanding of the frequency and types of overlap in healthcare utilization among autistic AYA. Such knowledge can inform whether changes should be made to the guidelines or clinical practices. We sought to begin to address such questions through studying The Center for Autism Services and Transition (CAST). A primary-care based medical home for autistic young adults.

CAST is a program based at The Ohio State University Medical Center in Columbus, Ohio (Hart et al., 2021; Saqr et al., 2018). CAST operates within a larger primary care practice that provides care to people of all ages, and so is well-positioned to meet the needs of autistic adolescents and young adults as they enter adult care. The program’s team and procedures adapt usual primary care practice to meet the unique needs of autistic AYA with particular attention to their transition to adult care as well as continuing to provide primary care for autistic adults into adulthood. As a primary care-based clinic, CAST is not equipped to make a new diagnosis of autism, but requires documentation of an existing autism diagnosis for patient eligibility. The only eligibility criteria for patients to be seen in CAST are documentation of an autism diagnosis and being between the ages of 16 and 26, so there is a wide range of differing abilities in patients seen at the CAST clinic. Some are seen independently, some have some visit time being seen alone and some with a caregiver present, and others require the assistance of a parent or caregiver for the entire visit (Hart et al., 2021). CAST has seen over 1000 autistic AYA since it was started in 2014. Many of the patients seen at CAST were referred to CAST from Nationwide Children’s Hospital, a large children’s hospital based in the same city and located about 15 min drive away. While some pediatric hospitals have established hospital-wide approaches to the transition to adult care for their patients (Hergenroeder et al., 2016), NCH has not. Nonetheless, because so many CAST patients have received care at both CAST and NCH, the study of health care use of patients seen at both institutions presents an important opportunity to understand the transition from pediatric to adult health care for autistic individuals.

In this study, we sought to characterize the amount and nature of continued pediatric care utilization for autistic youth who had established with an adult primary care physician to better understand the presence and nature of overlap of pediatric and adult care in this population. We specifically sought to answer three research questions:

1) What proportion of autistic AYA continue some kind of pediatric health care after their initial visit with an adult primary care doctor and how many visits are they having?

2) Among those continuing to receive pediatric health care after the first adult primary care visit, what types of care are they receiving?

3) Which patient characteristics are associated with continuing to obtain pediatric health care after the initial adult primary care visit?

## Method

### Study Design

This is a retrospective cohort study of deidentified electronic medical record (EMR) data.

### Patient Population

Patients included in this study had all been seen at least once at CAST between 4/1/2014 and 5/31/2020. Patients also needed to have at least one encounter at NCH prior to their first CAST visit but did not have to have NCH contact after their first CAST visit. We included all patients who met these visit criteria. No specific exclusion criteria were applied, as we wanted to include the broadest range of patients possible. As noted above, patients had to provide documentation to confirm the autism diagnosis prior to being seen at CAST. We did not reassess the diagnosis before including patients in this study. Because many patients seen at CAST were diagnosed prior to the advent of the DSM V severity criteria, we are not able to provide specific information regarding severity level with our data set.

This study was approved by the Nationwide Children’s Institutional Review Board with a waiver of consent approved based on the fact that the study involved deidentified EMR data. Community members were not involved in this study.

### Data Set Acquisition and Preparation

To acquire the data set, an honest broker (a trained data scientist outside the study team) was provided with a list of all CAST patients and then the electronic medical records of these patients were cross-referenced to patients seen at NCH, matching by name and date-of-birth. The EMR data of all patients for whom a match could be made were then pulled and deidentified. A deidentified data set of all EMR data for all matched patients (*n* = 244) was provided to the study team for the analyses, of which 230 people met all inclusion criteria and 14 did not. Those not meeting all inclusion criteria were excluded from the analysis. The study authors then extracted the relevant variables and constructed the measures for our analyses as described below.

### Measures

#### Determination of Being Established in Adult Care

For the purposes of this study, the first in-person visit to CAST with a physician was used as the marker of being established in adult care. We used this definition of being established because generally a first visit with a new primary care practitioner is considered to be established with that new practitioner, even as we recognize that this first visit establishing adult primary care is not the end of the transition process (Hart et al., 2019; White & Cooley, 2018).

#### Characterization of Pediatric Visits

Contact with NCH (the pediatric hospital) that occurred on or after the date of the first CAST visit was considered as overlap. We included a broad set of contacts between the family and a clinician at NCH in our definition of pediatric care received during the overlap period. We included encounters with a clinician in person, such as office encounters and hospital stays. We also included other types of encounters, such as phone calls, pharmacy visits, and any messages sent via the NCH patient portal. This was done to ensure a broad description of the contact that patients and families have with their pediatric team after establishing with an adult primary care clinician. All canceled and no-show appointments were removed, as were encounters where a patient left without being seen and any encounters that were not classified, which we determined were mostly administrative. An example of an encounter that was not classified was the travel screening obtained at registration prior to an appointment.

Encounters were categorized as either “direct patient contact” or “indirect patient contact.” Examples of direct patient contact include office encounters, hospital encounters, nurse visits, pharmacy visits, physical therapy sessions, and telemedicine visits. Examples of indirect patient contact include telephone encounters, nurse triage calls, and orders-only encounters. We also noted if the pediatric contact occurred before or after being established in adult care (i.e. before or after the first CAST visit).

#### Patient Characteristics

In addition to assessing pediatric visits, we evaluated the relationship between these eight patient characteristics and receiving overlapping care.

- Age (one characteristic)– we used age at first CAST visit to define participant age in this study. We included age in the analysis because older age is associated with greater odds of transfer to an adult provider (Jensen et al., 2017; Reid et al., 2004).
- Body Mass Index (BMI) (one characteristic) – we used BMI at first CAST visit to define participant BMI in this study. Because many of the medications being assessed are weight promoting, we felt that accounting for BMI in the adjusted analyses was important (Egan et al., 2013).
- Presence of an International Classification of Diseases, Tenth Revision (ICD-10) code for autism (F84.X, where X is zero to nine) on patient’s problem list at NCH (one characteristic). While all patients had to be diagnosed with autism to establish with CAST, this diagnosis was not always made within the NCH system, and so we thought it would be useful to determine if the NCH system had acknowledged the autism diagnosis within the EMR as part of our assessment of care overlap.
- Total number of pediatric encounters at NCH before their first CAST visit (one characteristic) – We determined the number of NCH visits prior to the first CAST visit for each patient, up to four years before the first CAST visit. We anticipated that significant involvement with NCH prior to the first CAST visit would be associated with continued involvement with NCH after the first CAST visit, and so wanted to assess for this relationship in our analysis.
- Medications (four characteristics) – The deidentified data set for the study included all medications that had been prescribed to all patients within the EMR. We chose to assess the relationship between psychiatric medication use and continuing in pediatric care after the first CAST visit because limited access to appropriate adult mental health care has been noted as barrier to transition to adult care for autistic people (Kuhlthau et al., 2016; Malik-Soni et al., 2022). To identify psychiatric medications from the medications prescribed in the EMR, the full medication list was pulled from the data set and then two authors (C.H. and L.H.) independently classified the medications into drug classes, which were then collapsed into higher-level groups to simplify the analyses. Drug class designation was based on how a medication is used in the care of autistic people. We opted for this approach as several of the medications on our list had off-label uses in autistic people that would not be captured by standard taxonomies for medications, such as the use of n-acetylcysteine for the treatment of disruptive behaviors in autistic people. Any discrepancies were adjudicated by discussion until consensus was reached. This classification process resulted in four groups of psychiatric medications, all of which were included in the analysis. They are:
  - Selective serotonin reuptake inhibitors (SSRIs) / selective norepinephrine reuptake inhibitors (SNRIs), which are used primarily for the treatment of depression and anxiety and include medications such as sertraline, fluoxetine, and venlafaxine.
  - ADHD medications, which included both stimulant and non-stimulant treatments for ADHD. An example of stimulant treatment for ADHD includes methylphenidate, and an example of non-stimulant treatment for ADHD includes atomoxetine.
  - Anti-psychotics, which included medications such as risperidone, aripiprazole, and clozapine. These medications treat a range of conditions from insomnia to psychosis and were grouped together due to working on a similar part of the brain, since it can be difficult to tell what the medicine was intended to treat when relying on EMR data.
  - Other psychiatric medications (such as benzodiazepines, doxepin, trazodone, and hydroxyzine). Any medications used for the treatment of psychiatric conditions and disruptive behavior in autistic people that did not fall into one of the above three categories was classified in the “other psychiatric medication” sub-group.

#### Demographics

The following demographic information was also extracted from the data set in order to provide a general descriptive information of the population: race (as self-reported in the EMR), insurance information (delineated as Medicare, Medicaid, and / or Private), and gender (as self-reported in the EMR). We also determined the proportion of patients with depression, ADHD, and anxiety by calculating the proportion of patients who had this documented on their problem list (list of ongoing chronic conditions) in the EMR, as these conditions commonly occur with autism and determining the proportion of patients with these conditions allowed for an opportunity to assess how our study population compared to autistic people generally. This information was not used in the analysis. Non-binary and transgender designations were not consistently documented in either hospital EMR during much of this study, and so this information was not available to us.

### Analysis

Significance was set at *p* ≤ .05 a priori for all analyses. We used complete case analysis for the logistic and linear regressions, meaning those with missing data were removed.

#### Study Aim 1: Examine the Proportion of Patients Getting Pediatric Care after the First Adult Appointment

For our first study question, we calculated the proportion of patients who had any kind of contact with NCH after the first CAST visit and the mean number of NCH visits among patients who had at least one NCH visit after the first CAST visit.

#### Study Aim 2: Assess the Types of Care Received after the First Adult Appointment

As noted above, we categorized all contact between patients and NCH as either direct or indirect. To describe the types of care received, we determined the proportion of patients who had direct contact, indirect contact, or both with NCH after their first CAST visit. We also determined the median number of direct and indirect encounters among those who had at least one contact with NCH after the first CAST visit.

#### Study Aim 3: Evaluate Patient Characteristics Associated with Continued Pediatric Contact

To assess which patient characteristics were associated with continued pediatric care, we chose three different analytic strategies: (a) comparison of those with no NCH contact after the first CAST visit to those with NCH contact after the first CAST visit, broken down by quartiles, (b) unadjusted (single variable) and adjusted (multi-variable) logistic regression to determine the odds of continued pediatric care after the first CAST visit, (c) unadjusted and adjusted linear regression to quantify the relationship between the selected characteristics and volume of continued pediatric care after the first CAST visit among those who had at least one contact with NCH after the first CAST visit. The first analysis provides initial impressions regarding the relationships between the characteristics and continued contact at NCH. The second allows for addressing any confounding of the relationship between having overlap or not (Stoltzfus, 2011). The third provides an opportunity to assess the relationship between the characteristics and volume of continued NCH contact, a continuous variable, among those who had some degree of contact, also controlled for any confounding that might be present, a question for which linear regression is well-suited (Grant et al., 2018).

For the comparison of characteristics by quartiles, we broke down the patients into sub-sets based on the number of overlapping pediatric NCH encounters the patient had after their first encounter with the CAST program. These subsets were: patients with no NCH encounters and patients with at least one NCH encounters (including both direct and indirect encounters) broken into quartiles, based on population proportion, for a total of five subsets. The quartiles were separated by number of NCH encounters: the first quartile was for patients that had one to three NCH encounters, the second quartile was for four to 13 NCH encounters, the third quartile was for 14 to 45 NCH encounters, and the last quartile was for 46 to 838 NCH encounters. We reported the aggregated statistics for each of the eight patient characteristics described above for each subset. Statistical significance was calculated with Pearson’s r or a t-test as appropriate.

We next completed a logistic regression (Stoltzfus, 2011). The outcome variable of the logistic regression was the odds of a patient having any post-CAST pediatric encounter. The initial logistic regressions were done as bivariable analyses to look at each patient characteristic separately (unadjusted analyses), determining the relationship between odds of having overlap and each characteristic. We also fit a multivariable logistic regression (adjusted analysis) to assess which characteristics remained significant after controlling for the other characteristics in the model. Patients were removed from the multivariable analysis if they had missing data, leaving 188 patients for the adjusted logistic regression.

We then performed a linear regression. The outcome variable of the linear regression was the number of pediatric encounters after the first CAST visit among those who had at least one pediatric encounter after the first CAST visit. Because everyone in this analysis had to have at least one NCH visit after the first CAST visit, 136 patients were included in the linear regression. Our first step in the linear regression was to compare each of the eight patient characteristics of interest serving as the independent variable in a bivariable (unadjusted) analysis and then to perform a multi-variable (adjusted) analysis with all eight patient characteristics included.

Of note, in both the logistic and linear regression models, the BMI and number of pre-CAST NCH encounters were transformed into log space to make the distributions of these independent variables more symmetric (i.e. closer to being normally distributed). In the linear regression model, the number of post-CAST NCH encounters was also log transformed.

## Results

### Patient Characteristics

Demographic characteristics of the 230 patients in this study are in Table 1, as are those of the patients included and not included in the logistic regression. For the full cohort, the mean age at the time of the last pediatric visit was 19.8 years, 82% were male, and 68% were White. About 10% of patients had co-occurring depression, 30% had co-occuring ADHD, and 26.1% had co-occuring anxiety.

#### Study Aim 1: Proportion of Patients Getting Pediatric Care After the First Adult Appointment

Out of the 230 patients that were included in this study, 149 (65%) had some kind of contact with the pediatric system after their first CAST visit (Table 1), with a median of 13 pediatric encounters after the first CAST visit. Our decision to exclude no show and cancelled visits removed five patients from the overlap group.

#### Study Aim 2: Types of Care Received After the First Adult Appointment

As shown in Table 1, over half (129 patients or 56%) had a visit involving direct patient contact after their first CAST visit. A substantial number (106 or 46%) had both direct patient contact and indirect patient contact with NCH after their first CAST visit. A smaller fraction (10%) had only indirect contact with the pediatric health system after the first CAST visit. The median number of pediatric encounters after the first CAST visit was 13 (IQR 42). The median number of direct encounters was four (IQR 15) and the median number of indirect encounters was seven (IQR 24).

#### Study Aim 3: Patient Characteristics Associated with Continued Pediatric Contact

Table 2 shows the patient characteristics comparing those with no pediatric contact after the first CAST visit to each quartile of pediatric care use after the first CAST visit. Patients had an increase in both direct and indirect encounters with each quartile. Younger age at the time of the first CAST visit and a higher number of pediatric encounters prior to the first CAST visit were significantly associated with having more pediatric encounters after the first CAST visit (*p* < .05 and *p* < .001 respectively). Use of psychotropic medications was common, but not significantly different across groups.

The results of the logistic regression can be found in Table 3. The final model had a pseudo R^2^ (goodness-of-fit value) of 0.1130, indicating that our model of eight variables does not adequately reflect the complexities of the occurrence of overlapping care. Given our relatively small sample size and simplification of phenomena into only eight characteristics, this result is not unexpected. In the logistic regression analysis of characteristics associated with odds of having a pediatric contact after the first CAST visit, those of an older age at the time of the first CAST visit had lower odds of having a pediatric encounter after first CAST visit in both the unadjusted and adjusted analyses (Table 3). In the adjusted analysis, the OR for age was 0.80, meaning that for each one-year increase in age, the odds of having overlap decreased by 20%. Prescriptions for psychiatric medications were associated with higher odds of a pediatric visit after the first CAST visit in the unadjusted analyses, but these were not statistically significant in the adjusted analyses. Conversely, the presence of one of the ICD-10 codes for autism on the pediatric problem list was not associated with a pediatric visit after the first CAST visit in the unadjusted analyses (OR 0.78, 95% CI 0.41–1.49) but was associated with lower odds of a pediatric contact after the first CAST visit in the adjusted analyses (OR 0.26, 95% CI 0.08–0.85).

Table 4 shows the results of the linear regression assessing for relationships between patient characteristics and number of pediatric visits after the first CAST visit. The final model had a Deviance of 14,451 (136 Observations, eight degrees of freedom) and a Pseudo R^2^ value of 0.1463, indicating that our model of eight variables does not adequately reflect the complexities of overlapping care frequency. Given our relatively small sample size and simplification of phenomena into only eight characteristics, this result is not unexpected. Age was negatively associated with number of pediatric visits after the first CAST visit in the adjusted and unadjusted analyses, meaning that those who were older had fewer pediatric visits after the first CAST visit than those who were younger. Number of pediatric encounters before the first CAST visit was positively associated with the number of pediatric encounters after the first CAST visit in both the unadjusted and adjusted analyses. Among the other characteristics assessed (BMI, psychiatric medication groups, presence of an autism diagnosis code on the problem list), none had significant relationships to the number of pediatric encounters after the first CAST visit in either the unadjusted or adjusted analyses.

## Discussion

In this study, a significant majority of patients had some kind of contact with the pediatric health system after their first visit to adult primary care, and over half had direct patient contact with a pediatric clinician after the first visit in adult primary care. We found that younger age and more pediatric encounters prior to the first adult appointment were associated with continued use of pediatric care after the first adult appointment. We also found that having an ICD-10 code for an autism diagnosis in the pediatric EMR was associated with lower odds of a pediatric encounter after the first CAST visit in the adjusted analyses.

### Study Aim 1: Proportion of Patients Getting Pediatric Care After the First Adult Appointment

Transition is a process, and while the steps may be clear and linear in the guidelines, they may not be clear for a particular patient’s life journey (Sezgin et al., 2020). In this study for example, the patients had established their adult primary care provider, but continued to get other services, such as specialty care, physical therapy, and pharmacy support from the pediatric hospital after that first adult primary care visit. Each component of a patient’s care is making the transfer to the adult system separately, and so the functional result is a portion of the transition process where patients and families are interacting with both the pediatric and adult health systems.

Nearly two thirds of patients had some kind of contact (either direct or indirect) with the pediatric health system after the first adult primary care appointment. This suggests that overlap in care is the norm rather than the exception for autistic adolescents and young adults when a flexible approach that uses factors other than age to determine the timing of transfer to adult care is in effect. There are many reasons this overlap may be occurring. Prior studies demonstrated that autistic adults reported frequent negative experiences with healthcare professionals in the past (Vogan et al., 2017). This may make autistic individuals hesitant to trust new providers and thus maintain relationships with pediatric providers longer until comfort develops with the adult care team. Caregivers of autistic youth have reported lacking information about the healthcare transition process and lack of communication between pediatrician and adult PCP as a barrier to care during the transition process (Kuhlthau et al., 2016). This may add to the desire to maintain prior healthcare relationships as a safety-net until they feel more established with their adult healthcare provider. Since guidelines and expert opinion encourage a flexible approach (Hardin & Hackell, 2017; Hart & Maslow, 2018; Schor, 2015; White & Cooley, 2018), the resulting overlap needs to be more explicitly accounted for in those guidelines, as well in clinical care, research, and policy, so that providers, scientists, payors, and administrators approach transition assuming that overlap will be present.

### Study Aim 2: Types of Care Received After the First Adult Appointment

Over half of patients had direct contact with a pediatric provider after their first adult primary care appointment. This finding raises questions about the use of “bounce backs” as a quality indicator for a successful transition, as has been done elsewhere (Gray et al., 2019; Sadun et al., 2022; Sharma & Sharma, 2022; Zupanc, 2020). With so many patients making use of pediatric care after the first adult primary care appointment in this study, use of such a measure will need to be carefully done. We acknowledge that someone returning to a particular pediatric specialist after the first appointment with the equivalent adult specialist may be a sign of a problem. For example, returning to the pediatric endocrinologist after the first adult endocrinology appointment for someone with diabetes may indicate an inadequate transition process. However, a return to the pediatric neurologist for headaches after the first adult endocrinology appointment may be appropriate, if the neurologist has opted to transfer to adult care at a different time.

### Study Aim 3: Patient Characteristics Associated with Continued Pediatric Contact

We found that younger age at the time of the first adult appointment and a larger number of pediatric visits before the first adult appointment were associated higher odds of overlap and that the presence of an ICD-10 code for autism in the medical record was associated with lower odds of overlap. None of the medication groups were associated with higher or lower odds of overlap.

One possible explanation for younger age being associated with higher odds of overlap is that more complex patients were intentionally scheduled with an adult primary care clinician at a younger age to allow for time to get all health needs met before fully exiting the pediatric system. If done thoughtfully, this kind of transition could allow patients and families to feel supported and may represent the correct approach: a slow move of care to the adult-oriented system over time to allow for adaptation to the new system to occur. Such a graded approach has been tested in other populations and been shown to be well-received (Chaudhry et al., 2013; Sheehan et al., 2015). However, other explanations should be considered. Autistic youth and their families have reported that they did not feel well-supported during the transition to adult care (Cheak-Zamora & Teti, 2015), and studies show that the basic components of transition are not being addressed regularly for autistic youth (Leeb et al., 2020). Thus, it is also possible that more complex autistic patients are being moved to adult care at a younger age without adequate preparation and then patients and families return to the pediatric health system in various ways as a reflection of that lack of adequate preparation.

We chose to evaluate psychotropic medication use as a possible predictor of overlap because lack of appropriate psychiatric / mental health support (i.e. willing providers with knowledge of the care needs of autistic people) has been identified as a barrier to transition to adult care for autistic people (Kuhlthau et al., 2016; Malik-Soni et al., 2022). However, in our study, there was no association between prescriptions for psychotropic medications and continued use of pediatric care in adjusted analyses. It is possible that, for CAST patients, a willing and knowledgeable primary care clinician was taking over prescribing of these medications, thus addressing this barrier to transition for this cohort. It’s also possible that reasons outside of mental health access are playing a role in the determination of timing of the transfer to adult health services. For example, our study found that the presence of an ICD-10 code for autism on the problem list was significantly associated with lower odds of a pediatric visit after the first CAST visit. Interpreting this finding is challenging, as there are likely many factors impacting documentation of a diagnosis on the problem list of an electronic medical record (EMR) system. It is possible that documentation differences reflect different clinical practice approaches by separate medical providers that may play a role in transition and transfer of patients. Prior studies show that even with robust EMR-based supports, implementation of transition practices is variable among medical providers (Harris et al., 2021). Our data set does not provide information about individual providers, so we are unable to assess this possible contributor. Further study will be needed to understand this relationship.

One commonly noted barrier to transition and transfer of care is lack of adult provider knowledge and comfort in caring for autistic patients (Kuhlthau et al., 2016). Our study evaluated transition of patient care to adult PCPs with a specific focus on the autistic population, so that effect may have been mitigated in our situation. However, when considering the health care transition needs of autistic patients, education of providers is an important consideration and ongoing effort to address this will be important. Recent work highlights one approach to this that resulted in increased provider comfort but, despite the increased comfort, noted that other barriers including lack of time for longer visits or to provide special accommodations within the clinic continued to present barriers to providing best-practice care (Mazurek et al., 2020). Thus, education and improving comfort of PCP’s alone may not be enough. Further efforts to address reimbursement and other administrative supports may be important for improving healthcare for autistic AYA.

### Limitations

This study has limitations. It is based on data from a cohort of patients seen at one children’s hospital and followed at one particular program for autistic adults. As such, it may not generalize to other groups. We feel it is telling, however, even in this relatively well-supported cohort, that overlap was so common. We suspect other patients with less organized systems who make the move to an adult provider may experience even more overlap between pediatric and adult care, particularly if those patients see several pediatric specialists. Additionally, we do not have DSM 5.0 data regarding autism severity. However, the rates of co-occurring mental health conditions are similar to those of autistic people generally (Lai et al., 2019). This suggests that, to the extent we are able to assess it, the population of the CAST clinic is representative of autistic people generally.

Patients were identified based on the fact that they had established with an adult primary care provider, and so data regarding those patients who were not able to get established in adult care are not considered here. As this study used retrospective EMR data, we do not have direct information from patients, families, or clinicians as to why the overlap in care occurred.

The racial and gender breakdown, while representative of autistic people in our area, makes it difficult to know if experiences differ by gender or race. Additionally, we only evaluated continued pediatric care after the first adult primary care appointment. Patients may have been receiving some adult specialty care before the first adult primary care appointment, which would have changed the numbers.

### Future Directions

The current study begins to look at the overlap of pediatric and adult care for autistic adolescents and young adults. Future work is needed to assess the timing of overlap and the reasons for overlap. We were not able to assess for any sort of interaction between age and complexity, though this interaction is a possible explanation of overlap that should be explored in future studies. Our models only explained a portion of the overlap, and so further study with larger groups of patients are needed to more completely understand the patient factors associated with overlap. Prescriptions for psychotropic medications were not related to overlap, but this finding may be a function of the unique environment of CAST and should be evaluated in other settings. Larger studies are also needed to evaluate possible differences in overlap among different racial groups and to assess differences by gender. Finally, this study informs future work to determine the appropriate measures of successful transition as the presence of overlap between pediatric and adult care should be accounted for when determining transition success.

**Table 1:** Demographic features of the Cohort

	Full Cohort (*N* = 230)		Included in the Logistic Regression (*n* = 188)		Not Included in the Logistic Regression (*n* = 42)	
	Avg/Count	IQR/%	Avg/Count	IQR/%	Avg/Count	IQR/%
Age at Last Pediatric Visit [Avg (IQR)]	19.8	3.3	19.8	2.8	19.6	6.2
Male Gender [Count (%)]	189	82.2%	153	81.4%	36	85.7%
Race [Count (%)]						
Black	36	15.7%	32	17.0%	4	9.5%
White	157	68.3%	121	64.4%	36	85.7%
Insurance [Count (%)]^a^						
Public (Medicare/ Medicaid)	181	78.7%	152	80.9%	29	69.0%
Private	155	67.4%	123	65.4%	32	76.2%
Patients with Pediatric Contact after first CAST visit [Count (%)]^b^	149	64.8%	136	72.3%	13	31.0%
Indirect Encounters Only	23	10.0%	21	11.2%	2	4.8%
Direct Encounters Only	20	8.7%	19	10.1%	1	2.4%
Both Direct and Indirect	106	46.1%	96	51.1%	10	23.8%
Median Number of Pediatric Encounters after first CAST visit [Med (IQR)]^c^	13.0	42.0	14.0	42.25	11.0	14.0

^a^Note that percentages add up to > 100% due to patients having multiple kinds of insurance

^b^Percentages here represent percentages of the total cohort for each column

^c^Among those with at least one pediatric encounter after the first CAST visit

**Table 2:** Patient characteristics by number of pediatric encounters after first CAST visit, by Quartile^a^

Pediatric Encounters after First CAST Visit	Zero	Quartile			
		First (1–3)	Second (4 to 13)	Third (14 to 45)	Fourth (46–838)
Total number of Patients (*n* = 230)	81	43	32	37	37
Mean age at first CAST visit^c^ [Avg (std)]	21.2 (5.6)	20.0 (2.1)	19.1 (2.8)	18.9 (2.6)	17.7 (2.7)
Mean BMI at first CAST visit [Avg (std)]	26.8 (8.2)	27.6 (8.1)	27.7 (8.0)	27.8 (7.6)	25.7 (6.2)
*Pediatric Encounters after first CAST visit [Avg (std)]*					
All pediatric encounters	0 (0.0)	1.6 (0.8)	7.8 (3.2)	26.3 (9.3)	149.1 (148.3)
Indirect pediatric encounters	0 (0.0)	0.8 (0.7)	3.5 (3.0)	10.4 (7.9)	47.5 (53.9)
Direct pediatric encounters	0 (0.0)	0.9 (0.9)	4.3 (2.5)	16.0 (7.5)	101.6 (105.8)
Pre-CAST pediatric encounters^b^	52.5 (75.1)	104.2 (95.2)	94.8 (95.3)	169.8 (140.4)	160.4 (183.2)
Presence of F84.X (ICD-10 codes for autism) on the pediatric problem list [% of Quartile]	79.0	79.1	68.8	86.5	73.0
*Medications [% of Quartile]*					
Prescribed an anti-psychotic [%]	42.0	65.1	59.4	75.7	48.7
Prescribed an SSRI/SNRI [%]	50.6	53.5	53.1	48.7	64.9
Prescribed a medication for ADHD [%]	39.5	69.8	62.5	64.9	59.5
Prescribed other psychiatric medications [%]	51.9	72.1	71.9	67.6	51.3

^a^Quartiles determined among those with at least one pediatric encounter after the first CAST visit

^b^Significantly associated with overlap in care (*p* < .001)

^c^Significantly associated with overlap in care (*p* < .05)

**Table 3:** Unadjusted and adjusted odds ratios comparing patient characteristics with attendance at a pediatric encounter after the first CAST visit

Patient Characteristic	Unadjusted analyses			Adjusted analysis		
	Odds Ratio	95% CI	N	Odds Ratio	95% CI	N
Age at first CAST visit	**0.79** ^a^	(0.71, 0.89)	227	**0.80** ^a^	(0.69, 0.93)	188
BMI at first CAST visit^d^	1.33^b^	(0.42, 4.18)	189	1.37^b^	(0.39, 4.84)	188
Presence of F84.X (ICD-10 codes for autism) on the pediatric problem list	0.78^c^	(0.41, 1.49)	230	**0.26** ^c^	(0.08, 0.85)	188
Prescribed an anti-psychotic in pediatric care	**2.23** ^c^	(1.25, 3.99)	230	0.77^c^	(0.31, 1.92)	188
Prescribed an SSRI/SNRI in pediatric care	**2.06** ^c^	(1.13, 3.75)	230	0.94^c^	(0.43, 2.04)	188
Prescribed a medication for ADHD in pediatric care	**2.58** ^c^	(1.43, 4.63)	230	1.44^c^	(0.66, 3.14)	188
Prescribed other psychiatric medications in pediatric care	**2.67** ^c^	(1.45, 4.89)	230	2.21^c^	(0.95, 5.17)	188
Number of pre-CAST NCH encounters^d^ (Max 4 years prior to first CAST visit)	**1.48** ^b^	(1.20, 1.83)	230	1.21	(0.89, 1.63)	188

^a^OR here represents the change in probability when age increases by 1

^b^OR here represents the change in probability when independent variable doubles

^c^OR here represents the change in probability when the patient characteristic is present

^d^Variable has been log-transformed

**Table 4:** Unadjusted and adjusted comparisons between patient characteristics and number of pediatric encounters after the first CAST visit*

Patient Characteristic	Unadjusted analysis			Adjusted Analysis		
	Coef	p-value	N	Coef	p-value	N
Age at first CAST visit	**-0.2089**	**< 0.001**	148	**-0.1684**	**0.004**	136
BMI at first CAST visit*	-0.5569	0.30	137	-0.4118	0.44	136
Presence of F84.X (ICD-10 codes for autism) on the pediatric problem list	-0.2685	0.40	149	-0.2039	0.57	136
Prescribed an anti-psychotic in pediatric care	-0.0964	0.73	149	0.3948	0.30	136
Prescribed an SSRI/SNRI in pediatric care	-0.0319	0.91	149	-0.1591	0.62	136
Prescribed a medication for ADHD in pediatric care	-0.3432	0.22	149	-0.3953	0.23	136
Prescribed other psychiatric medications in pediatric care	-0.4357	0.12	149	-0.3952	0.27	136
Number of pre-CAST pediatric encounters* (Max 4 years prior to first CAST appointment)	**0.1620**	**0.01**	149	**0.1772**	**0.009**	136

*Numbers log transformed for analytic purposes

## Abbreviations

AYA: adolescents and young adults
ADHD: attention-deficit hyperactivity disorder
CAST: Center for Autism Services and Transition
EMR: electronic medical record
BMI: body mass index
ICD-10: International Classification of Diseases, Tenth Revision
SSRI: selective serotonin reuptake inhibitor
SNRI: selective norepinephrine reuptake inhibitor
NCH: Nationwide Children’s Hospital
