# Correction: Continuation of Pediatric Care after Transfer to Adult Care Among Autistic Youth Overlap of Pediatric and Adult Care

**DOI:** 10.1007/s10803-024-06393-4

**Published:** 2024-06-22

**Authors:** Joseph Sirrianni, Christopher Hanks, Steve Rust, Laura C. Hart

**Affiliations:** 1 Nationwide Children’s Hospital, 700 Children’s Drive, Columbus, OH 43205 USA; 2 The Ohio State University College of Medicine, Columbus, OH USA

**Correction: Journal of Autism and Developmental Disorders**

10.1007/s10803-024-06314-5

In the original article, a section of the introduction contains error

“The Center for <<name redacted for anonymity> >CAST,”

The correct one should read as follows, “The Center for Autism Services and Transition (CAST).”

This has been corrected in this article.

